# Supplementary material for: Characterization of Ocular Surface Microbial Profiles Revealed Discrepancies between Conjunctival and Corneal Microbiota
Source: Pathogens. 2021 Mar 30;10(4):405. doi: 10.3390/pathogens10040405 (PMC8067172; doi:10.3390/pathogens10040405)
Supplement: Supplementary file 1 [file pathogens-10-00405-s001.pdf]

Characterization of ocular surface microbial profiles revealed discrepancies between conjunctival and corneal microbiota

Pathogens

Anna Matysiak[1,*] Michal Kabza[1*], Justyna A. Karolak[1,2], Marcelina M. Jaworska[1], Malgorzata Rydzanicz[3], Rafal Ploski[3], Jacek P. Szaflik[4], Marzena Gajecka[1,2]

[1]Chair and Department of Genetics and Pharmaceutical Microbiology, Poznan University of Medical Sciences, Poznan, Poland
[2]Institute of Human Genetics, Polish Academy of Sciences, Poznan, Poland
[3]Department of Medical Genetics, Medical University of Warsaw, Warsaw, Poland
[4]Department of Ophthalmology, Medical University of Warsaw, Warsaw, Poland
*These authors contributed equally to this manuscript

Corresponding author:
Marzena Gajecka, Ph.D.
gamar@man.poznan.pl

Table S1: Characteristics of ocular microbial flora depending on the applied methods

**References**	**Yearof test**	**Material**	**Number of subjects**	**Microbial groups** (not all detected microbial groups in subjects are included)								
				***Staphyloccus* spp.**	***Streptococcus* spp.**	***Propionibacteriu m* spp.**	***Nocardia/ Actinomyces* spp.**	***Acinetobacter* spp.**	***Escherichia* spp.**	***Pseudomonas* spp.**	***Corynebacterium* spp.**	**Fungi**
				**Microbiota identified using standard microbiology cultures [% of all detected microbial groups][a]** Percentage of presence chosen microorganisms based on published data and results of our examination.								
Capriotti et al.	2009 [11]	CS[b]	276	48.5	nd[b]	nd	6.5	nd	2.5	6.2	nd	26
Doan et al.	2016 [6]	CS	428	47.4	3.5	33.9	nd	nd	0.2	nd	15.4	1.1
Eder et al.	2005 [12]	CS	190	42	8	20	nd	nd	4	nd	16	2
Ozkan et al.	2017 [9]	CS	129	46.5	<5	34.9	nd	nd	nd	<1	6.2	nd
Martins et al.	2004 [15]	CS	60	73.4	nd	ne[b]	ne	nd	nd	3.4	38.4	nd
Fernández-Rubio et al.	2010 [13]	CS	4597	44	9	12	ne	nd	<1	<1	26	ne

				**Microbiota identified using PCR techniques [% of subjects][a]** Percentage of subjects in which chosen microorganisms based on published data were detected.								
Wu et al.	2003 [16]	CS	30	ne	ne	ne	ne	ne	ne	ne	ne	23
Wu et al.	2003 [16]	COR[b]	32	ne	ne	ne	ne	ne	ne	ne	ne	38
				**Microbiota identified using16S rRNA sequencing [% of subjects][a]** Percentage of subjects in which chosen microorganisms based on published data and results of our examination were detected.								
Dong et al.	2011 [8]	CS	4	2	0.5	nd	8	9	nd	18	11	na[b]
Ozkan et al.	2017 [9]	CS	43	0.2	4.8	nd	nd	11	nd	10.4	11.1	na
Ozkan et al.	2019 [28]	LM[b]	20	1.8	0.3	nd	nd	nd	nd	65.1	7.6	na
Ozkan et al.	2019 [28]	CS	45	3	2.3	nd	nd	present	nd	2.1	4	na
Wen et al.	2017 [40]	IBC[b]	90	95	12	100	nd	46	68	24	nd	na
Graham et al.	2007 [14]	CS	16	81	nd	19	nd	nd	nd	nd	19	na
				**Microbiota identified using16S rRNA sequencing [% of the detected genera][a]** Percentage of all detected genera in samples.								
Zhou et al.	2014 [2]	CS	220	3	5	4	nd	nd	nd	nd	16	na

Huang et al.	2016 [10]	CS	31	5.28	2.85	nd	nd	4.74	nd	26.75	28.22	na

[a]The various methods were applied in published data, so table is divided into section reffering to percentages of all detected microorganisms in traditional culture technique or all samples qualified to examination in molecular techniques. In 16S rRNA sequencing additionally there are percentage of the all detected genera in examinations. Using various names (% of positive culture, examined subjects, detected genera, total sequences etc.) to show results may lead to difficulties in creating a unified table.

[b]Abbreviations in table: COR-cornea, CS-conjunctival swab, IBC- inferior bulbar conjunctiva, LM-lid margin, nd-not detected, ne-not examined, na- no data

Table S2: Specific primers used to detect particular microorganisms in the conjunctival samples

Microorganisms	Primer Oligonucleotide sequences (5′-3′)	Amplicon size (bp)
Staphylococcus spp.	F: GTGAATCTGTTTGGCAGGTC<br>R: AAACGGATGGTGAACGAACT	443
Staphylococcus aureus	F: GGTGATGCTAAAACGATTGC<br>R: GCATTTCGGTGCTTACCTTT	231
Staphylococcus epidermidis	F: TTGAGCTTGTCATTGGTTCG<br>R: TGTAGAGGTTGCACGTCGAG	581
	F: ATCAAAAAGTTGGCGAACCTTTTCA<br>R: CAAAAGAGCGTGGAGAAAAGTATCA	124
Staphylococcus pasteuri	F: CCAATAGAACCAATAACAGGAAT<br>R: ATGTGGAGCAGTTAAGAGAA	157
Staphylococcus capitis	F: GCTAATTTAGATAGCGTACCTTCA<br>R: CAGATCCAAAGCGTGCA	208
Staphylococcus warneri	F: TGTAGCTAACTTAGATAGTGTTCCTTCT<br>R: CCGCCACCGTTATTTCTT	63
Staphylococcus haemolyticus	F: GTTGAGGGAACAGAT<br>R: CAGCTGTTTGAATATCTT	85
Escherichia spp.	F: ATCGTTCGTGGTTCTGCTCT<br>R: CACCTACGTTCTCACCAGCA	322
Escherichia coli	F: GTGCTTATCCCGGTCGTTTA<br>R: GGATCGGAAGTCATGTGGTT	158
Streptococcus spp.	F: AGGGGCTAGGTGAAATGGAC<br>R: CGCTGGGGACCTACGTATTA	629
Streptococcus pneumoniae	F: TAGAACGCTGAAGGAGGAGC<br>R: TAATACGTAGGTCCCGAGCG	494
Propionibacterium spp.	F: GGTGATACGTAGGGTGCGAG<br>R: CTTCCGGTACGGCTACCTTG	997
Propionibacterium acnes	F: AACCGCTTTCGCCTGTGA<br>R: ACGCTCAGGGTTAAGCCC	182
Ureaplasma spp.	F: ATCTCAAAAATAGCTTCATGATTGTG<br>R: CGGTAAGCAAATTATTTTTAATCGTT	201
Chlamydia spp.	F: GGATGTCCAAGATAAATATTACAGACAA<br>R: CTCGTTTCACACCTAGAGAACATAATAG	400
Mycoplasma spp.	F:<br>GAGGGGAGTGAAATAGATTCTGAAACCA	900

	R: AGTACAGGAATATTAACCTGTTGTCCATCG	
Moraxella catarrhalis	F: CCCATAAGCCTGACGTTAC<br>R: CTACGCATTTCACCGCTACAC	237
	F: GTGAGTGCCGCTTTACAACC<br>R: TGTATCGCCTGCCAAGACAA	71
Pseudomonas spp.	F: GACGGGTGAGTAATGCCTA<br>R: CCACTGGTGTTCCTTCCTATA	616
Acinetobacter spp.	F: CGGCTTTTTAAGTCGGATGTGA<br>R: CTTTCGTACCTCAGCGTCAGTA	184
Micrococcus spp.	F: CTTAACTCTGGGATAAGCCTGG<br>R: AATCTTTCCAACACCCACCA	80
Candida spp.	F: TCCGTAGGTGAACCTGCGG<br>R: TCCTCCGCTTATTGATATGC	520
Candida albicans	F: TTTATCAACTTGTCACACCAGA<br>R: ATCCCGCCTTACCACTACCG	273
Candida parapsilosis	F: GCCAGAGATTAAACTCAACCAA<br>R: CCTATCCATTAGTTTATACTCCGC	297

Table S3: The questionnaires data analysis

	**corneal samples donor [%]**	**conjunctival samples donor [%]**
Female	46	61
Male	54	39
18-35 years old	64	67
36-60 years old	36	33
General illnesses including allergies, diabetes, atopic dermatitis, heart disease	64	61
Ocular illnesses	100	44
Eye rubbing	20	17
Uncomfortable ocular symptoms	undefined	33
Reading books and working in front of computer (above 8 hours/day)	undefined	17
Using optic medicines	undefined	17
Using systemic medicines	undefined	39
Travelling	undefined	50
Smoking	20	17
Ocular surgeries	100	5

Regular ophthalmic examination	100	33
